# Thirtieth Anniversary of the Brazilian Journal of Cardiovascular Surgery. And Devising the Next Decades

**DOI:** 10.21470/1678-9741-2017-0501

**Published:** 2017

**Authors:** João de Deus e Brito, Walter J. Gomes, Paulo Roberto B. Evora, Domingo M. Braile

**Affiliations:** 1 Faculdade de Medicina da Universidade Federal do Rio de Janeiro, Rio de Janeiro, RJ, Brazil.; 2 Escola Paulista de Medicina, Universidade Federal de São Paulo (UNIFESP), São Paulo, SP, Brazil.; 3 Department of Surgery and Anatomy da Faculdade de Medicina de Ribeirão Preto da Universidade de São Paulo (FMRP-USP), Ribeirão Preto, SP, Brazil.; 4 Faculdade de Medicina de São José do Rio Preto (FAMERP), São José do Rio Preto, SP, Brazil and Universidade de Campinas (UNICAMP), Campinas, SP, Brazil.

During all year of 2016, we were commemorating the 30^th^ anniversary of the Brazilian Journal of Cardiovascular Surgery (BJCVS). We asked some collaborators of the Journal to express their sentiments about the interaction of them with the publication, and the importance that it represented in their lives.

Was not surprise to have many important manifestations of the colleagues, to show historical facts and personal impressions about the challenge that the former "Revista Brasileira de Cirurgia Cardiovascular" faced since its foundation up to nowadays.

The BJCVS was a dream of the members from the "Sociedade Brasileira de Cirurgia Cardiovascular" (SBCCV) that is supporting the publication along all years by a decision of all the associates. Even with sacrifice, are maintaining the Journal, without any reimbursement, from the authors, or from the readers.

From the very beginning, we had the possibility to count with the manuscripts from the Brazilian Cardiovascular Surgeons, offering the best products of the researches done in Brazil, to be published in our own Journal.

To grow in the very restrict field of the specialty, was not easy. However, with the total compromise of all collaborators was possible, step by step, to arrive at the 30^th^ anniversary in a consolidated position, publishing seven editions per year, six conventional and one supplement.

Being recognized by Main International Databases, like Medline - PubMed, PubMed Central, SCImago - Scopus, Thomson Reuters, and others, is a reason of great proud. Today we have the privilege to be in a good position in the Latin American Journals ranking in our field, where we are surpassed only by the sister organization "Arquivos Brasileiros de Cardiologia" See at the link: https://goo.gl/Kkiuxg

Our efficient Associate Editor, Prof. Dr. Paulo Evora, wisely decided to bring part of those emotional homages to thanks the authors, and give the opportunity to the readers to revive part of our history.

In the context of the celebrations of the 30^th^ anniversary of the Brazilian Journal of Cardiovascular Surgery, its Editor (Domingo M. Braile) invited associated surgeons to write about their personal feelings about the journal's history. Three texts were selected for a special session published in BJCVS 31.3 (2016). However, due to editorial standards, Scientific Electronic Library Online (SciELO) did not post those "historical" manifestations, which can only be read through the discontinued publication system, which has recently replaced by the Scholar One system. Curiously, without any previous consult, they were complementary in preserving the memory of the BJCVS.

## Historical notes (João de Deus e Brito)

The BJCVS had it birth place in our Society and had been a strong historical trajectory of uninterrupted publications along these 30 years. It became the unique international issuance in the field of the Southern hemisphere, Mexico, including Caribbean countries.

It was founded by the idealism led by Prof. Dr. Adib Jatene (*in memoriam*), who became the first Editor-in-Chief for ten years: 1986-1996. He had taken the first step by indexing in Latin American and Caribbean Health Sciences Literature (LILACS), and it was an important starting point.

Prof. Dr. Fabio B. Jatene was the second Editor-in-Chief, between 1986 and 2002, establishing the first electronic version, by indexing in SciELO, and introducing the peer system.

Prof. Dr. Domingo M. Braile, in 2002, made himself available and had been dedicated permanently, as well as his predecessors, to the fate of Journal since then. His main point is consecutive fights for renewal and improvement of the Journal, only compared to cut a rough diamond. After many years of fights, MEDLINE indexing was finally announced on October 31^st^, 2007.

During the celebrations of 25 years, in 2011, we had the first Impact Factor (IF) published by ISI-Thomson Reuters, the prime number of 0.963, almost 1, at first evaluation, which shows the high degree of development of Brazilian Cardiovascular Surgery.

After indexing in the most relevant databases, possessing one the most qualified Editorial Board who works passionately, it was impossible to go ahead. Some changes were done to meet the needs of a new international reality. In this way, we changed the name of the Journal (BJCVS), modified the submission and evaluations electronically, which became fully in English. As a result, we started receiving articles from others ten countries worldwide, such as the United States of America, Turkey, and China, etc. As a result the publications increased from quarterly to bimonthly, in 2015. We have broken what the Editor-in-Chief commonly called the "nostalgia to remain unread by the international community" for a long time^[1]^.

Remarkable technical innovations of our surgeons and foundation of cardiovascular surgery centers in all Brazil had been relevant in the development and increasing number of publications along the last 30 years. We should not take for granted this accomplishment, but we continued to fight to be among most prestigious international publications of the specialty.

My thanks to the honorable invitation and congratulations to the Editor-in-Chief and his predecessors, presidents of our Society and everyone who contributed along these 30 years of the state-of-the-art of our BJCVS. Certainly, this Journal will continue in the future to come of new generations of cardiovascular surgeons worldwide. In all we have made a good choice.

## Technical and editorial notes (Walter J. Gomes)

In recent decades the Brazilian cardiovascular surgery has achieved milestones, an extraordinary evolution occupying a prominent position in the international scenario, with a significant increase in the volume of surgical procedures, development of new surgical techniques and a local industry that supported this rise.

Similarly, the union of cardiovascular surgeons enabled the construction of a robust and thriving specialty society, which has served as a model for other guilds over the country by the vigor in defense of their rights, achievements and attained leadership.

In the wake of these journeys, 30 years ago the BJCVS was launched, consolidating its position as the official journal for promoting the scientific production of the Brazilian cardiovascular surgeons. With strong leadership working hard as editors, continuous developments and changes contributed to the journal reached this position. From the early conquests with the inclusion in SciELO portal allowing the electronic version in Portuguese and spread on the Internet to the listing and availability in the CTSNet gateway, the Internet page of cardiovascular surgeons from around the world.

The indexing in Medline/PubMed, the largest scientific database on the planet in health sciences, represented the maturity of the publication project and confidence in the evolution of quality.

But crucial decisions were made about the way to go, after long and exhaustive analysis. With globalization and the consequent need for dissemination of its scientific content, the adoption of the English language was natural and imperative to achieve this goal. Breaking the paradigm of a regional publication evolving to show up on the world stage. The change of the name to Brazilian Journal of Cardiovascular Surgery also stood for a challenge, with the scope to internationalize the journal and places it to assume a more representative position in the cardiovascular arena. English language translation or editing service has been provided for authors who are not fluent in this idiom, assisting in preparing a publication-ready.

Just like the other periodicals, the BJCVS strive to publish important and high-quality original scientific papers, which will serve for further interest in citation and therefore be increasing the impact factor. The impact factor ranks the scientific excellence of the periodicals worldwide, and all the efforts are focused on reaching the quality level attained by the top publications in the field.

And the adoption of new software of electronic submission, the Scholar One is intended to facilitate to local and overseas authors the straightforward presentation of the manuscript, avoiding delays and expediting the publication.

The publication has been expanded to six issues a year and the continuing medical education (CME) is provided in every issue, where the editor picks the article and write the related multiple choice questions.

Expanding the scope, the BJCVS also has served for publication of original works from multidisciplinary or multi-professional production, specialties and professionals of related areas working along cardiovascular surgeons and making possible achieve the high standard of healthcare and scientific excellence.

Seven associate editors have been assigned to assist in every sub-area of the cardiovascular surgery with the review of different types of manuscripts, select reviewers, review reports, and make the final decision to accept, reject, or revise. Moreover, a new team at the editorial office with Meryt Zanini as the Managing Editor and Camila Sáfadi taking over the position of Editorial Manager, greatly assisting and facilitating the work of the Chief Editor, the Associate Editors, and the reviewers. And all of them led by Professor Domingo Braile, the enthusiastic and tireless Chief Editor, whose hard work has enabled to reach this stage.

Rearrangements of the journal's sessions will permit to cover articles on Innovations and New Technologies and keep *pari passu* with the transformations the specialty.

After all, several reasons make attractive to publish in BJCVS a relevant article. A journal with 30 years of tradition, indexed in PubMed / Medline which consequently allows the authors greater exposure of their paper, besides the free access on the Web to the full content of the articles and free of charge.

But BJCVS still have to climb higher levels, complete the internationalization process and get to be next to the best journals in the world.

## Academic and Philosophical notes (Paulo Roberto B. Evora)

"Brazil is much greater than the crises it faces. It is essential to show what we are capable of in order to stand amidst other nations". (Domingo M. Braile, 2016)

Domingo Braile (BJCVS Editor) wrote: "We have a constant obsession with having a better impact factor and, as a result, a Qualis ranking compatible with our specialty, will suffer another drawback: due to the change of name, the BJCVS citation will be only in English and thereby counted by Thomson and Scopus from now on. These are problems to be faced in order to improve for the future"^[1]^. I think that our 0.526 impact factor, despite all our efforts, it is not compatible with the BJCVS Thomson Reuters evaluation. Maybe this is the only reason for sadness during the BJCVS 30 years celebration. However, some considerations may alleviate this feeling.

Randy Schekman, a US biologist who won the 2013 Nobel Prize in Physiology or Medicine receiving his prize in Stockholm, said his lab would no longer send research papers to the toptier journals, Nature, Cell and Science. Schekman said pressure to publish in "luxury" journals encouraged researchers to cut corners and pursue trendy fields of science instead of doing more important work. The problem was exacerbated, he said, by editors who were not active scientists, but professionals who favored studies that were likely to make a splash. Writing in the Guardian, Schekman raises serious concerns over the journals' practices and calls on others in the scientific community to take action. "I have published in the big brands, including papers that won me a Nobel prize." But no longer, he writes. "Just as Wall Street needs to break the hold of bonus culture, so science must break the tyranny of the luxury journals." A journal's impact factor is a measure of how often its papers are cited and is used as a proxy for quality. But Schekman said it was "toxic influence" on science that "introduced a distortion." He writes: "paper can become highly cited because it is good science - or because it is eye-catching, provocative, or wrong"^[2]^.

Although Dr. Sheckman's piece has received a fair share of criticism, including remarks of obvious hypocrisy, it does bring attention to certain emerging problems within the scientific community. One primary concern, which Sheckman touched upon, is the widespread use of the impact factor as a measure of research quality and productivity. The impact factor was originally established by Thomson Reuters Corporation as a tool to help librarians identify journals to purchase^[3]^. However, when applied by funding agencies, academic institutions, and other parties, the impact factor is often used as the primary parameter to evaluate an individual's or an organization's scientific contributions. Meanwhile, scientists alike agree that the impact factor does not appropriately measure the quality of a scientific article nor does it reflect how influential the work is in the field^[3]^. Thus, in recognizing the misleading influence of the impact factor, scientists across various disciplines are voicing a call for change.

Finally, it would be appropriate to highlight some points that are involved directly and indirectly with the impact factor: a) High taxes for publications; b) The so-called "research consortium" with papers that have up to 200 co-authors; c) Often abusive self-citation rates, and; d) The billionaires news editorial groups mergers, etc. For our society, the impact factor does not matter. So, Dr. Braile we do not have to ask "for whom the bell tolls, we are not an island, the bell tolls for our BJCVS 30 years" (Jon Donne)...
